# Development and in-vivo validation of a portable phosphorescence lifetime-based fiber-optic oxygen sensor

**DOI:** 10.1038/s41598-023-41917-5

**Published:** 2023-09-07

**Authors:** Lilian Witthauer, Emmanuel Roussakis, Juan Pedro Cascales, Avery Goss, Xiaolei Li, Alexis Cralley, Dor Yoeli, Hunter B. Moore, Zhaohui Wang, Yong Wang, Bing Li, Christene A. Huang, Ernest E. Moore, Conor L. Evans

**Affiliations:** 1 Wellman Center for Photomedicine, Massachusetts General Hospital, Harvard Medical School, Charlestown, MA 02129 USA; 2 Department of Diabetes, Endocrinology, Nutritional Medicine and Metabolism, Inselspital, Bern University Hospital and University of Bern, 3010 Bern, Switzerland; 3 Department of Surgery, University of Colorado Denver/Anschutz Medical Campus, Aurora, CO USA

**Keywords:** Organic chemistry, Photochemistry, Hypoxia, Applied optics, Biochemistry, Health care, Optics and photonics, Cardiovascular diseases, Trauma, Engineering, Biomedical engineering

## Abstract

Oxygenation is a crucial indicator of tissue viability and function. Oxygen tension ($\hbox {pO}_2$), i.e. the amount of molecular oxygen present in the tissue is a direct result of supply (perfusion) and consumption. Thus, measurement of $\hbox {pO}_{{2}}$ is an effective method to monitor tissue viability. However, tissue oximetry sensors commonly used in clinical practice instead rely on measuring oxygen saturation ($\hbox {StO}_2$), largely due to the lack of reliable, affordable $\hbox {pO}_2$ sensing solutions. To address this issue we present a proof-of-concept design and validation of a low-cost, lifetime-based oxygen sensing fiber. The sensor consists of readily-available off-the shelf components such as a microcontroller, a light-emitting diode (LED), an avalanche photodiode (APD), a temperature sensor, as well as a bright in-house developed porphyrin molecule. The device was calibrated using a benchtop setup and evaluated in three in vivo animal models. Our findings show that the new device design in combination with the bright porphyrin has the potential to be a useful and accurate tool for measuring $\hbox {pO}_2$ in tissue, while also highlighting some of the limitations and challenges of oxygen measurements in this context.

## Introduction

Oxygen sensing is a vital aspect of medical care, providing important information about a patient’s respiratory and cardiovascular function as well as tissue viability. Oxygen tension, also known as partial pressure of oxygen ($\hbox {pO}_2$), can vary depending on the specific tissue and its metabolic activity. For example, arterial blood presents a $\hbox {pO}_{{2}}$ of 75–100 mmHg^1,2^ while for skeletal muscle it is in the range of 5–90 mmHg^3,4^. The liver’s $\hbox {pO}_2$ values have been reported to have a wide range and appear to be influenced by the physiological condition. For example, liver transplant recipients have shown higher $\hbox {pO}_2$ levels of 60 mmHg two days after the transplant, whereas intraoperative $\hbox {pO}_2$ values obtained using a different approach have recorded a median $\hbox {pO}_2$ level of 31 mmHg^5,6^. In general values vary depending on factors such as inspired oxygen, exercise, and disease states.

Tissue viability is dependent on maintaining sufficient oxygen levels, with oxygen deprivation leading to tissue damage and, potentially, necrosis. Therefore, real-time, direct measurement of oxygen tissue concentration could be particularly helpful in the diagnosis and management of various conditions such as compartment syndrome^4,7–10^, ischemia and reperfusion injuries^11^, microvascular disease in people with diabetes^12^, organ perfusion during shock, hemorrhage, sepsis^13^, flap reconstruction^14^ and wound healing^15–17^.

The use of the first oxygen sensors in medicine dates back to the late 19th century^18^. One of the earliest and most widely used oxygen sensors was the Clark electrode^19^, developed by Leland Clark in the 1950s. This sensor uses an electrochemical reaction to measure $\hbox {pO}_2$ in a gas or liquid sample. The Clark electrode has been widely used in a variety of applications, including oxygen sensing in medical settings, industrial processes, and environmental monitoring. Phosphorescence-based oxygen sensors have begun to emerge as an alternative to electrochemical sensors^20–23^, a shift motivated by a number of factors, including the relative fragility and cost of Clark electrodes. Phosphorescence-based sensors utilize the oxygen-dependent quenching of phosphorescence of certain materials, such as metalloporphyrins, to measure oxygen levels^2,24–30^, and they have the advantage of requiring less maintenance while providing high signal stability. Later, fiber-optic technology has been incorporated into phosphorescence-based oxygen sensors (e.g.,^31–33^), enabling the development of compact sensors with high sensitivity and accuracy. Despite the availability of commercial laboratory oxygen sensors for in vitro and in vivo applications (e.g. PreSens in Germany and OxyLite by Oxford Optronix in the United Kingdom) and significant research efforts to develop miniaturized oxygen-sensing fiber probes^34–49^ and corresponding in vivo experiments^50–53^, none have been approved for use in humans as of yet. Therefore, by advancing a portable prototype equipped with enhanced porphyrin molecules, sensor formulation, and sensor construction, our objective is to facilitate $\hbox {pO}_2$ monitoring in surgical settings.

In this research paper, we present a highly sensitive, fiber-based oxygen sensor designed for use in vivo that leverages a brightly emitting, in-house developed metalloporphyrin phosphor^54^ measured via a compact and portable all-in-one phosphorescence lifetime approach. The bright metalloporphyrin emission enables strong signals from minute concentrations of porphyrin, and has been employed across a wide array of medical applications^10,17,55–59^. In contrast to a previous study that employed a phosphorescence intensity-based setup^10^, the measurement of the oxygen concentration in this case is based on the measurement of lifetime, which presents several major advantages including improved signal, device, fiber, and motion stability. The small footprint of the device, its ability to operate completely stand-alone, and its ability to run off battery power makes it an advancement in the field of fiber-based oxygen sensors. The developed sensor was validated in a set of bench and in vivo experiments. Our results demonstrate the potential of this fiber-based oxygen sensor to be a valuable tool for $\hbox {pO}_2$ sensing in medical care and highlight some limitations of fiber-based oxygen sensors.

## Results

### Lifetime extraction

The lifetime was extracted from the detected phase shift between the sinusoidal reference signal and the phosphorescent signal via the equation:

1 $$\begin{aligned} \tau = tan(\Delta \theta )/(2\pi f_r) \end{aligned}$$

With $\Delta \theta =\theta _p-\theta _r$, i.e. the phase difference between the reference sinusoidal signal driving the LED ($\theta _r$) and the phosphorescence signal measured by the photodiode, and $f_r$ the reference frequency.

**Figure 1:**
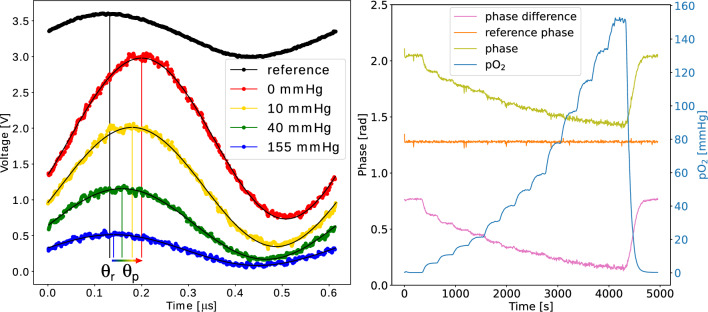
Left: voltage signal for different levels of $\hbox {pO}_2$. Right: phase signal as a function of time for varying values of $\hbox {pO}_2$.

The corresponding signals were acquired as follows. First, the LED was turned off and the background signal (time and voltage) was sampled 100 times with the analog-to-digital converter (ADC), with the mean value calculated. The LED was then pulsed at a frequency of $f_r=$1625 Hz using the corresponding Pulse width modulation (PWM) pin. The resulting signal (time and voltage) was sampled 500 times, which was a trade-off between LED-on time (150 ms) and accuracy of the phase shift. The previously acquired background signal was then subtracted online during data acquisition from the signal. Using the modulo operator, one period with $T= 1/f_r$ of the reference signal and the phosphorescence signal was extracted as shown in Fig. 1. The phase of the reference ($\theta _r$) and phosphorescence signal ($\theta _p$) was calculated from these signals using a simple linear regression in matrix form^55,60^ based on the following equation:

2 $$\begin{aligned} y(t) = \beta _0 + \beta _{1} cos(2\pi f_r t) + \beta _{2} sin(2\pi f_r t) \end{aligned}$$

yielding a phase of

3 $$\begin{aligned} \theta = \arccos {\left( \frac{\beta _1}{\sqrt{\beta _1^2+\beta _2^2}}\right) } \end{aligned}$$

Due to improved filtering of the LED signal the fitting of the fundamental frequency yielded sufficient accuracy and no further odd harmonics needed to be considered as it was done in prior studies^55^.

As can be seen in Fig. 1, the phase difference as well as the intensity of the phosphorescence signal increases with decreasing levels of $\hbox {pO}_2$ due to decreased phosphorescence quenching.

### Calibration

The calibration of the oxygen sensing fibers was performed using an in-house developed test chamber connected to an automated gas mixer (IGM, Geometrics). The total gas flow was kept constant at 200 ml/min and the oxygen levels were adjusted by mixing different ratios of nitrogen and air. The oxygenation change step size was set depending on the $\hbox {pO}_2$ range: below 20 mmHg a step size of 5 mmHg was chosen, between 20 and 60 mmHg a step size of 10 mmHg was used and above 60 mmHg a step size of 20 mmHg was found to be reasonable. For each $\hbox {pO}_2$ step the gas ratios were kept constant for 5 min, which was the time needed for the chamber to reach equilibrium. As a reference sensor the OxyLite Pro (Oxford Optronix; UK) was used and $\hbox {pO}_2$ was measured every 3 s.

The humidity of the gas mixture was set to the maximum possible level of 90% allowed by the system in order to mimic the humid conditions in tissue. However, as shown in reference^10^, the oxygen sensing formulation is humidity insensitive, meaning that the oxygen sensing formulation yields the same reading of $\hbox {pO}_2$ independent of the level of water vapor.

The temperature in the gas chamber was adjusted using a water bath and a hot plate. Each oxygen sensing fiber was calibrated at three different temperatures: at room temperature, at 30 $^\circ$C, and around body temperature at 36 $^\circ$C. This allowed for the construction of a temperature-compensated calibration so the sensors can be used across the physiological temperature range.

**Figure 2:**
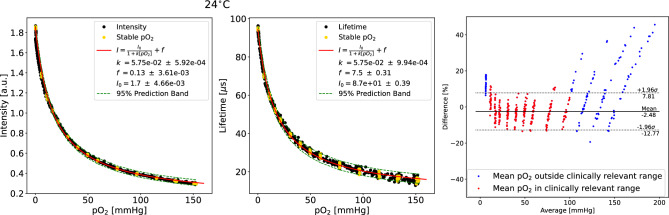
1D calibration for the sensor from the intensity and the lifetime signal (left two plots) at 24 $^{\circ }$C. A Bland–Altman plot for the lifetime-based extraction is shown on the right.

In Fig. 2, a lifetime calibration curve at room temperature is compared to the corresponding intensity calibration curve. Further calibration curves at 30 and 36 $^\circ$C are shown in Fig. S4. The 95% prediction interval is indicated by a green line and shows that the resolution for the lifetime extraction method was not as high as in intensity-based measurements, especially at high $\hbox {pO}_2$ levels. This is not surprising; while the intensity-based approach can integrate over time continuously, the lifetime based approach makes use of a single sampling frequency of 1625 Hz. The single frequency approach was selected to optimize the device for in vivo human applications in the physiological range, where high resolution oxygen measurements are rarely needed for clinical decision-making and low weight, simple operation, small footprint, and low operational power are key requirements. Depending on the operational range of $\hbox {pO}_2$ required, the selected frequency can be shifted: higher values optimize the sensing of higher oxygenation values when lifetimes are short while lower values optimize low oxygenation conditions. 1625 Hz was chosen as a balance between these extremes such that the accuracy within the clinically relevant range between 10 and 100 mmHg was found to be approximately $\pm \,10\%$, adequate for the majority of medical needs. This is also visible in the Bland-Altman plot in Fig. 2.

It should be noted that future devices could, as is done in many fluorescence lifetime measurements, sweep across a range of frequencies for improved accuracy. This, however, would come at the cost of longer acquisition times and greater photobleaching. While some degree of photobleaching can be seen in these experiments, it was observed to be relatively low and did not impact the accuracy of the lifetime-based measurement. This is important, as photobleaching has been observed to affect intensity based measurements, introducing changes to the maximum possible phosphorescence detected that must be tracked and require compensation. Lifetime measurements, as demonstrated here, operate essentially independently from photobleaching as long as the detected intensity is above a minimum signal-to-noise threshold.

**Figure 3:**
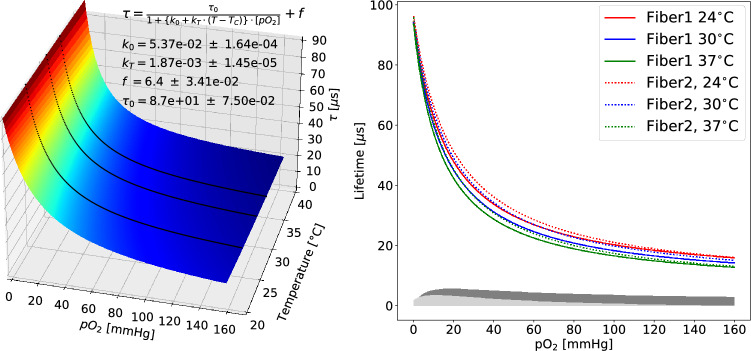
Left: 2D calibration for the sensor from the lifetime signal. Right: 1D calibration curve for two different fibers at three different temperatures each. The absolute difference between the two fibers at 24 $^\circ$C is shown as a light gray shaded area, the maximum difference induced by the temperature is shown as a dark gray shaded area.

The temperature and $\hbox {pO}_2$ dependent data was fitted with a temperature-compensated version of the Stern–Volmer equation^10,61^:

4 $$\begin{aligned} \tau = \frac{\tau _{0}}{1+(k_{0}+k_{T}\cdot (T-T_{C}))\cdot [pO_{2}]} + f \end{aligned}$$

where $\tau$ and *T* are the measured lifetime and temperature respectively, *f* accounts for the non-oxygen dependent phosphorescent background, $k_T$ is the temperature dependent quenching constant, and $T_C$ the room temperature at which the calibration was performed. A resulting two-dimensional calibration curve with the extracted parameters is shown in Fig. 3. In contrast to prior intensity-based devices^10^, the calibration of the present device was only slightly dependent on temperature and the fiber-to-fiber variations were minor (the difference is indicated by the shaded areas in the plot).

### In vivo intramuscular measurement

The portable lifetime-based sensor was tested in an in vivo porcine tourniquet model to determine its performance. The sensor was inserted into the flexor carpi ulnaris muscle in the front limb of a previously anesthetized pig. According to the standard procedure for pigs at Massachusetts General Hospital, anaesthesia was induced with intramuscular Telazol (4.4 mg/kg) and Atropine (0.4 mg/kg). The anaesthesia was continued with an isoflurane (1–3%) inhalation with a fraction of inspired oxygen ($\hbox {FiO}_2$) of 1. A commercial sensor (OxyLite Pro, Oxford Optronix) was tested alongside the prototype sensor.

**Figure 4:**
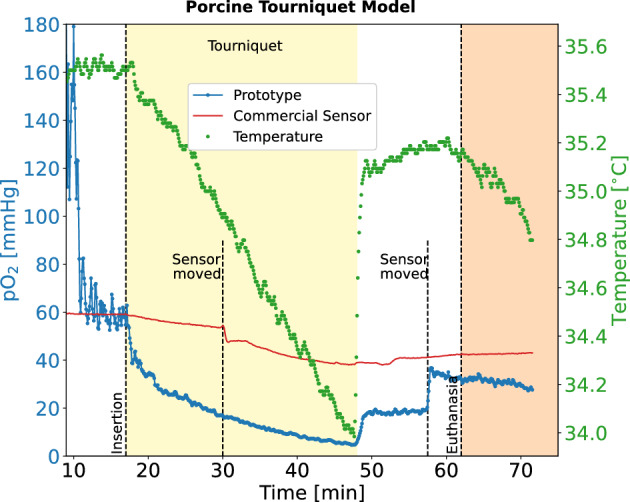
In vivo results from the pig tourniquet model. Blue: prototype sensor, red: commercial sensor (OxyLite), green: temperature from the thermocouple inside the prototype sensor.

The results from this in vivo study are shown in Fig. 4. After the prototype sensor equilibrated to approximately 65 mmHg, a tourniquet was applied to the limb. The prototype sensor showed an immediate decrease in $\hbox {pO}_2$, with the values decaying continuously to approximately 5 mmHg. The drop in oxygenation occurred alongside a measured drop in tissue temperature, read out via the thermocouple within the probe tip, indicating the successful placement of the tourniquet and subsequent loss of perfusion. After approximately 30 min, the tourniquet was released and the prototype sensor showed an instant recovery to approximately 20 mmHg, alongside a concomitant rise in temperature. Repositioning of the prototype into a different region of the limb showed an additional recovery to 40 mmHg. This behaviour of the oxygen tension matches the that found in our previous study using the same animal model^10^, including the lack of full recovery after tourniquet release, indicating possible acute tissue damage. The initial $\hbox {pO}_2$ value in the muscle of 60 mmHg is in agreement with results by Doro et al. in canine models^4^ but significantly lower than our previously measured 120 mmHg^10^. The differences can be explained by the heterogeneous oxygen distribution within the muscle (location and depth dependence) or by inter-individual differences between animals. After euthanasia, the $\hbox {pO}_2$ exhibited a gradual decay, consistent with findings from a related experiment conducted by our group^10^. It is postulated that the slow decay may arise from the inhalation of pure oxygen during anaesthesia.

We note that the commercial sensor did not show any appreciable change in $\hbox {pO}_2$ during these measurements, even after being moved to a second location approximately 30 min into the experiment; it is possible that during the insertion the needle used for the OxyLite sensor became clogged, or the sensor was inserted into a blood pool.

### Experiments with soft tissue animal models

In order to further assess the usability of the oxygen sensing prototype, additional experiments were performed with different soft tissue animal models at the University of Colorado School of Medicine. Since the OxyLite sensor proved not to be reliable in simple settings in animal models as well as in laboratory settings, it was not used for the these more complex soft tissue measurements.

#### In vivo dismounted complex blast injury model

In the second experiment, a combat casualty relevant dismounted complex blast injury (DCBI) swine model^62^ was used.

The DCBI model combines hemorrhagic shock (HS) and tissue injury (TI) with a blast traumatic brain injury (bTBI). Healthy adolescent male Yorkshire swine were acclimated for a minimum of 3 days, and weighed between 45 and 58 kg. Anesthesia was induced with ketamine (20 mg/kg), xylazine (2 mg/kg), and acepromazine (0.2 mg/kg) followed by intubation. Continuous infusion anesthesia using propofol (3 mg/kg/h) and fentanyl (3 mcg/kg/h) was maintained throughout the experiment.

Fixed pressure hemorrhagic shock was initiated by bleeding from 12 Fr femoral arterial catheters. The target mean arterial pressure (MAP) within 10 min was 20 mmHg. Once 20 mmHg was obtained, blood removal is titrated to maintain a MAP of 15 mmHg and an EtCO2 of 20 mmHg. Surgical cutdown through the skin and quadriceps of the extended hind limbs was done until the femur is located. A captive bolt stunner (Blitz-Kerner, Turbocut JOBB GmbH, Germany) was placed directly on each femur and confirmed with visualization. The bTBI was created with a Friedlander type blast wave using a compressed gas mobile shock tube. The mobile shock tube was housed in a outdoor 52 foot trailer and managed by Applied Research Associates, Inc. (Littleton, CO). A NIJ Level II vest, ear plugs, and goggles were placed on the swine in the operative room to limit unwanted blast exposure to the torso, eyes, and ears.

**Figure 5:**
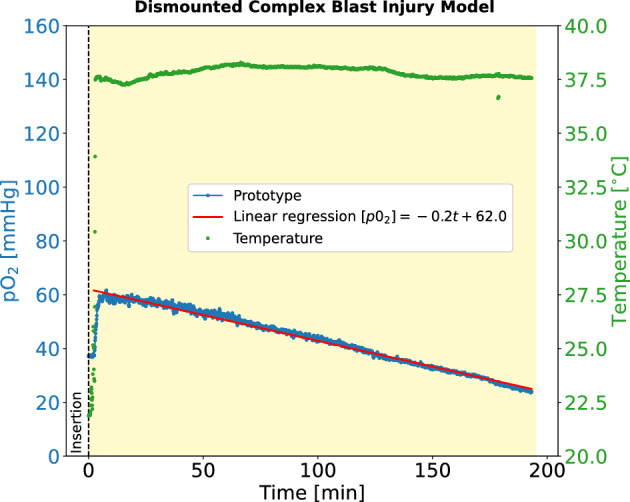
Hepatic $\hbox {pO}_2$ measured in an in vivo porcine dismounted complex blast injury model.

Subsequently, a laparotomy was performed on the animal, exposing the organs within the peritoneum. The prototype sensor probe was inserted directly into the liver tissue (right liver lobe) which was exposed through the surgical incision. As can be seen in Fig. 5, after an initial fast increase to approximately 60 mmHg, $\hbox {pO}_2$ declined steadily with a rate of 0.2 mmHg per minute over the next 3 h until euthanasia (determined by a linear regression). The initial value of 60 mmHg is above physiological levels in the liver of 30–50 mmHg reported in literature^63^ suggesting an increased influx of arterial blood and/or decreased metabolic demand due to tissue damage. The following decline in hepatic $\hbox {pO}_2$ was consistent with the severe shock produce by bleeding.

#### Rat hind limb transplant

In the third experiment, the oxygenation within a Brown Norway rat hind limb was monitored before and after syngeneic orthotopic transplantation to a recipient animal. All rats were placed under isofluorane anesthesia for the duration of the procedure. The protocol involved one rat that served as a donor for the hind limb and a second recipient animal that received the transplant. Donor and recipient hind limbs were retrieved by a circle around skin incision, ligation of epigastric vessels, microdissection of femoral vessels, and the donor leg was amputated at mid-femoral level with transection of the femoral vessels proximally to the inguinal ligament. The femoral artery was flushed with 5 ml of cold heparinized Ringer’s lactate solution and stored at 4 $^{\circ }$C. The recipient leg was amputated similarly but with transection of the vessels more distally than the donor. Osteosynthesis was performed using an 18-gauge needle for intramedullary fixation. Microsurgical anastomosis of the femoral artery and vein were performed with interrupted 10-0 nylon sutures.

**Figure 6:**
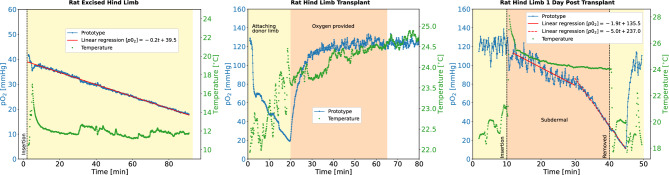
In vivo results from the excised donor rat limb (left), the hind limb during the transplantation (middle) and one day after the transplant (right). Shown is the oxygen from the prototype sensor in blue, the temperature in green, and a linear regression in red.

The oxygen sensing fiber was placed subdermally within the transplanted hind limb. After excision, the prototype oxygen sensor recorded a continuous drop in partial pressure of oxygen down to approximately 20 mmHg in the subcutaneous tissue with a slope of 0.2 mmHg/min (extracted from a linear regression), as shown in Fig. 6 (left). During this measurement and until being transplanted, the excised limb was preserved at low temperature by being placed over ice, the tissue temperature was around 12 $^\circ$C. The time period between the first two measurements (end of the excised limb monitoring and beginning of the measurement once re-attached) was approximately 1.5 h. The procedure to transplant the limb to the recipient animal took approximately 20 min during which the oxygenation changed significantly due to changes in perfusion and exchange with room air due to limb movement, as can be seen in Fig. 6 (middle). After the procedure, pure oxygen with 2.5% isofluorane was provided by means of a nose cone to the recipient animal at a rate of 3.5 l/min, which led to a slow increase of the limb oxygenation to approximately 120 mmHg over 30 min. The value of 120 mmHg can likely be explained by the inhalation of pure oxygen during anaesthesia. Due to the nature of the lifetime-based measurement, the prototype sensor had a single measurement precision of $\pm \,20$ mmHg at high $\hbox {pO}_2$ values. We note that the high $\hbox {pO}_2$ values measured in this case are due to the artificially high inspired oxygenation conditions, which would not typically be used in human healthcare settings. In addition, the material properties of the oxygen sensing layer might have changed during the low temperature in the tissue. One day after the transplant procedure, a measurement of oxygenation was repeated in the hind limb (Fig. 6, right). In this second day measurement, bleeding was observed alongside a decline in oxygenation readings (a linear regression led to a oxygen decline of 1.9 and 5 mmHg per minute, which is much steeper than the decay measured in the other in the above in vivo models); upon removal of the oxygen sensing fiber it was concluded that the two openings within the catheter had become filled, leading to an essentially clogged device. This observation suggests further device improvements that can be made for devices utilized under pro-inflammatory conditions such as post-surgical monitoring. Possibilities are larger needle openings or a saline flushing system to prevent clogging as well as improved coatings (e.g. heparin) to prevent spoilage.

## Discussion

This study demonstrated the development process and validation of a phosphorescence-based fiber optic oxygen sensor designed specifically for in vivo medical applications. The lifetime extraction method used in this study showed good sensitivity in measuring oxygen concentration across the physiological range. The method used in this study was not affected by photobleaching, a common issue with intensity-based measurement techniques. The sensor was calibrated at three different temperatures and the resulting data was fitted with a two-dimensional curve to account for temperature dependence. The lifetime signal was found to be far more stable against changes in temperature as compared to the intensity-based prototype of an earlier study^10^. In addition, the signal variation between different fibers was less pronounced than previously reported^10^. While the overall oxygen sensing resolution was found to be lower than in prior intensity-based devices, the device resolution was adequate for medical diagnostic needs and could be improved in next-generation devices using a sweep or range of reference frequencies.

To show the device’s diversity of in vivo applications, experiments were conducted to validate its performance in both pig and rat models. While the performance was overall found acceptable, it was noted that the small hole size used in the catheters in this study may be prone to clogging in soft tissues, especially under post-surgical and pro-inflammatory conditions. Clogging was interestingly not observed in muscle tissue. These observations have provided key insight for improved, clog-free designs that can be implemented in future development efforts.

Overall, the results of this study suggest that the developed oxygen sensing fiber probe with a bright oxygen sensing dye has the potential to be a valuable tool for monitoring oxygen tension with increased accuracy in physiological and pathological conditions, including compartment syndrome, reperfusion injuries, and flap reconstruction.

Future work will focus on testing the fiber probe in additional in vivo models and optimizing the probe design for first-in-man clinical application. Such additional measurements will be important to test updated iterations of the device as well as enhance the assessment of uncertainty and variability associated with the measurements. In vivo animal studies, in both healthy and diseased systems, will also provide important additional information on how to prevent problems with oxygen measurements in next-step clinical studies.

## Methods

### Oxygen sensing coating

The oxygen sensing fiber tip coating was prepared using a method previously described in^10^.The porphyrin can readily be synthesized using previously published protocols^54^ and formulated for use on the fiber^10^.

Poly(n-propyl methacrylate) (PPMA) was obtained from Scientific Polymer Products and dissolved in dichloromethane at a concentration of 0.025 mg/$\upmu$l by vortexing. The pivaloyl-terminated porphyrin was synthesized according to the method described in^54^ and added to the same tube containing the PPMA solution to reach a final concentration of 50 $\upmu$M.

### Fiber preparation

Before coating, the pig-tailed side of the 200 $\upmu$m multimode fiber (Thorlabs, FP200URT) was cleaned, stripped, and cleaved according to the method described in^10^. The fiber was then inserted, along with an ultrafine temperature microprobe (Physitemp IT-24P), into a flexible catheter (Braintree scientific SUBL 220). The fiber was secured in place using UV-curing glue (Loctite AA 3321) inside the catheter, with the tip protruding approximately one mm from the front of the catheter.

**Figure 7:**
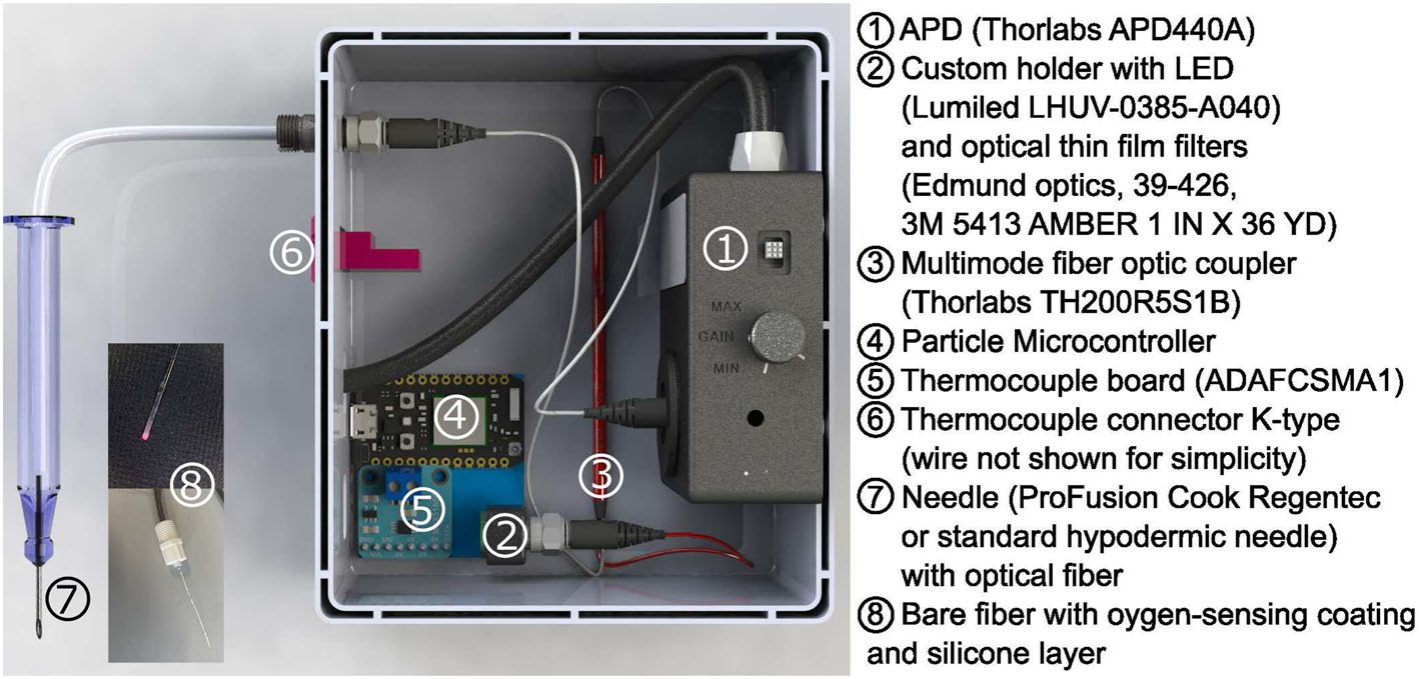
Device design showcasing electronic and optical components, accompanied by an enlarged view of the fiber tip.

To coat the fiber, 5 $\upmu$L of the previously prepared PPMA-porphyrin solution was transferred to a flat piece of a polydimethylsiloxane (PDMS) film. After a waiting period of 5–10 s, the fiber tip was dipped into the oxygen-sensing solution. The fiber was then left to dry overnight and placed in a high vacuum for 3 h to remove any remaining solvent. Finally, as described in^10^, the fiber was coated with a white scattering silicone/titanium dioxide layer to increase signal strength and reduce background contributions. A photo of the fiber and the coating are shown in Fig. 7.

Instead of the custom-made needles described in^10^, the fiber was inserted into a commercial needle with sideports arranged in a spiral around the distal end of the needle (ProFusion Cook Regentec). The sideports had a diameter of 0.56 mm, which was smaller than in the custom design previously used^10^.

### Prototype design

#### Optical components

The prototype design is shown in Fig. 7. Oxygen concentration-dependent phosphorescence was measured using a commercially available variable gain avalanche photo diode (APD, Thorlabs, APD440A) with a 1 mm diameter active area. The gain was set to the maximum value to prevent saturation of the APD by the porphyrin emission signal under zero oxygen (100% nitrogen) conditions. The porphyrin was excited by light from a light emitting diode (LED) with a peak wavelength of 385 nm (Lumiled LHUV-0385-A040). The LED was mounted on a printed circuit board in a custom 3D printed LED holder (Fig. S2). One fiber from the multimode fiber optic coupler (Thorlabs, TH200R5S1B) was connected to the LED holder using a glued SMA bulkhead adaptor (Thorlabs, HASMA) and the LED light was focused on the fiber tip using a glass bead (Winsted Precision ball 3/32 diameter). Between the glass bead and the SMA connector, several ultra-thin filters (Edmund optics 35-126, 35-127, 35-124) were stacked to serve as a 400 nm short pass to optimize the excitation spectra (see Fig. S1). The other side of the fiber optic coupler was connected to the APD via an adapter plate (Thorlabs, SM05SMA) and a flexible thin film filter (Edmund optics, 39-426) in combination with a polyamide film (3M 5413 AMBER 1 IN X 36 YD) was used to block any UV excitation light (Fig. S1). The remaining fiber from the fiber optic coupler was connected to a fiber optic mating sleeve (Thorlabs, ADAFCSMA1) mounted in the wall of the box.

#### Electronic circuit

The driver and readout circuits were designed analogously to those described in^55^. The intensity of the excitation LED was modulated using a sinusoidal driving voltage. The base for this signal was the square wave generated at the PWM output of the microcontroller. This square wave was then converted to a sinusoidal wave using a series of low pass filters (4-pole) and the voltage was amplified using a transimpedance amplifier circuit as described in^64^.

For current experiments, a reference frequency $f_r$ of 1625 Hz was found to provide an optimal balance between lifetime resolution and LED brightness. This reference frequency was achieved by using resistors of R = 980 $\Omega$ in the filter circuit ($f = 1/(2\pi RC)$ with $C=0.1\,\upmu$F). The APD was driven by its own independent temperature-compensated electronic circuit and power supply. The signal from the APD as well as the reference signal were sampled using a 12-bit analog-to-digital converter IC (ADC, Texas Instruments, ADS7828EIPWRQ1) that was connected to the microcontroller (particle, photon) via the I2C interface. A stable 5 V reference voltage for the ADC was provided by a booster converter (Texas instruments, TPS61099YFFR) circuit yielding a ADC resolution of 1.2 mV/bit. An I2C thermocouple amplifier board from Adafruit (MCP9600) was used to sample temperature. The full PCB schematics are shown in Fig. S3 and all optical and electronic components are summarized in Table S1. The total costs of the components is approximately 1700 USD, which is considerably lower than the costs of commercial devices such as the OxyLite system.

### Ethics approval

The animal study protocols were approved by the Institutional Animal Care and Use Committees (IACUC) of Massachusetts General Hospital (MGH) and the University of Colorado Denver under protocol IACUCU Nr. 2019N000231, IACUC Nr. 01050, and 00851. Both IACUCs are accredited by AAALAC. Department of Defense funded animal studies were also approved by the Animal Care and Use Review Office (ACURO) under protocol Nr. RT190072P1.e001. All studies were performed according to US federal regulations (Animal Welfare Act and Animal Welfare Regulations) and the guidelines by the Office of Laboratory Animal Welfare (OLAW) of the National Institutes of Health. All methods were reported according to ARRIVE guidelines.

### Supplementary Information

Supplementary Information.

## Data Availability

The data and materials presented in this study are openly available in Zenodo at https://doi.org/10.5281/zenodo.7568650.
