# Supplementary material for: Development and in-vivo validation of a portable phosphorescence lifetime-based fiber-optic oxygen sensor
Source: Sci Rep. 2023 Sep 7;13:14782. doi: 10.1038/s41598-023-41917-5 (PMC10484954; doi:10.1038/s41598-023-41917-5)
Supplement: Supplementary file 1 — Supplementary Information. [file 41598_2023_41917_MOESM1_ESM.pdf]

Supplementary Information to:

Development and In-Vivo Validation of a Portable Phosphorescence Lifetime-Based Fiber-Optic Oxygen Sensor

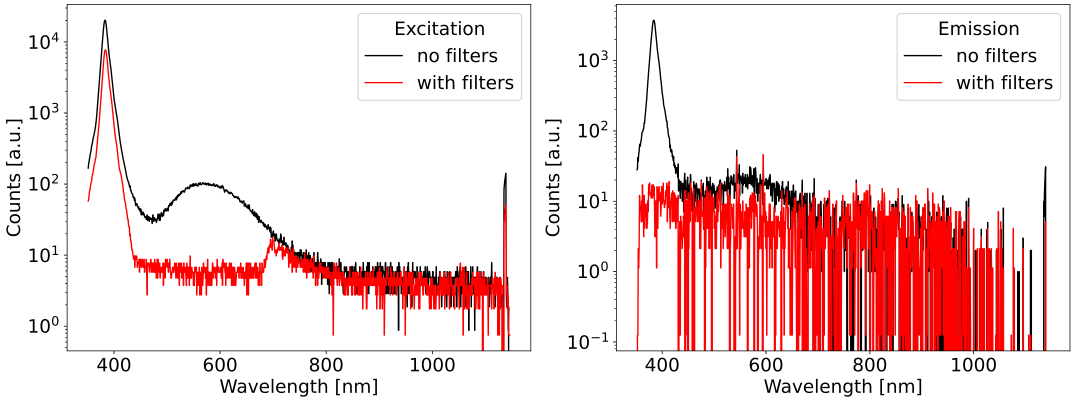

Figure S1: Excitation (left) and emission (right) spectra with and without filters.

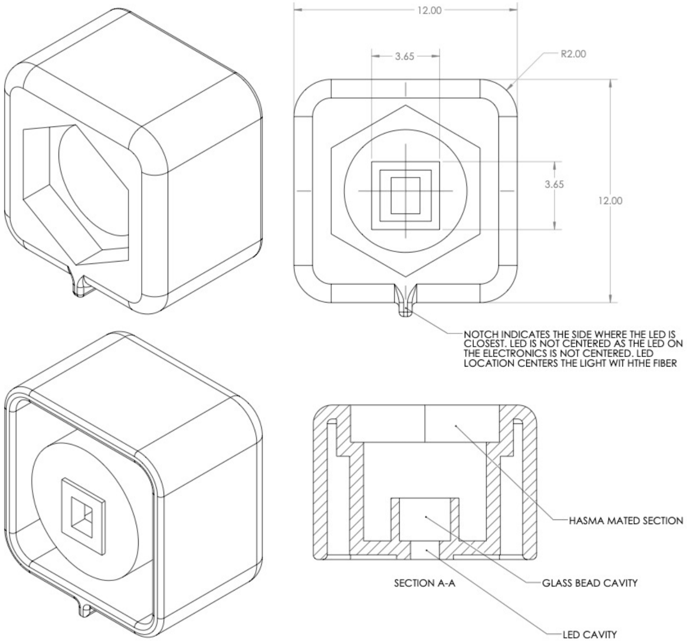

Figure S2: Custom-designed LED holder.

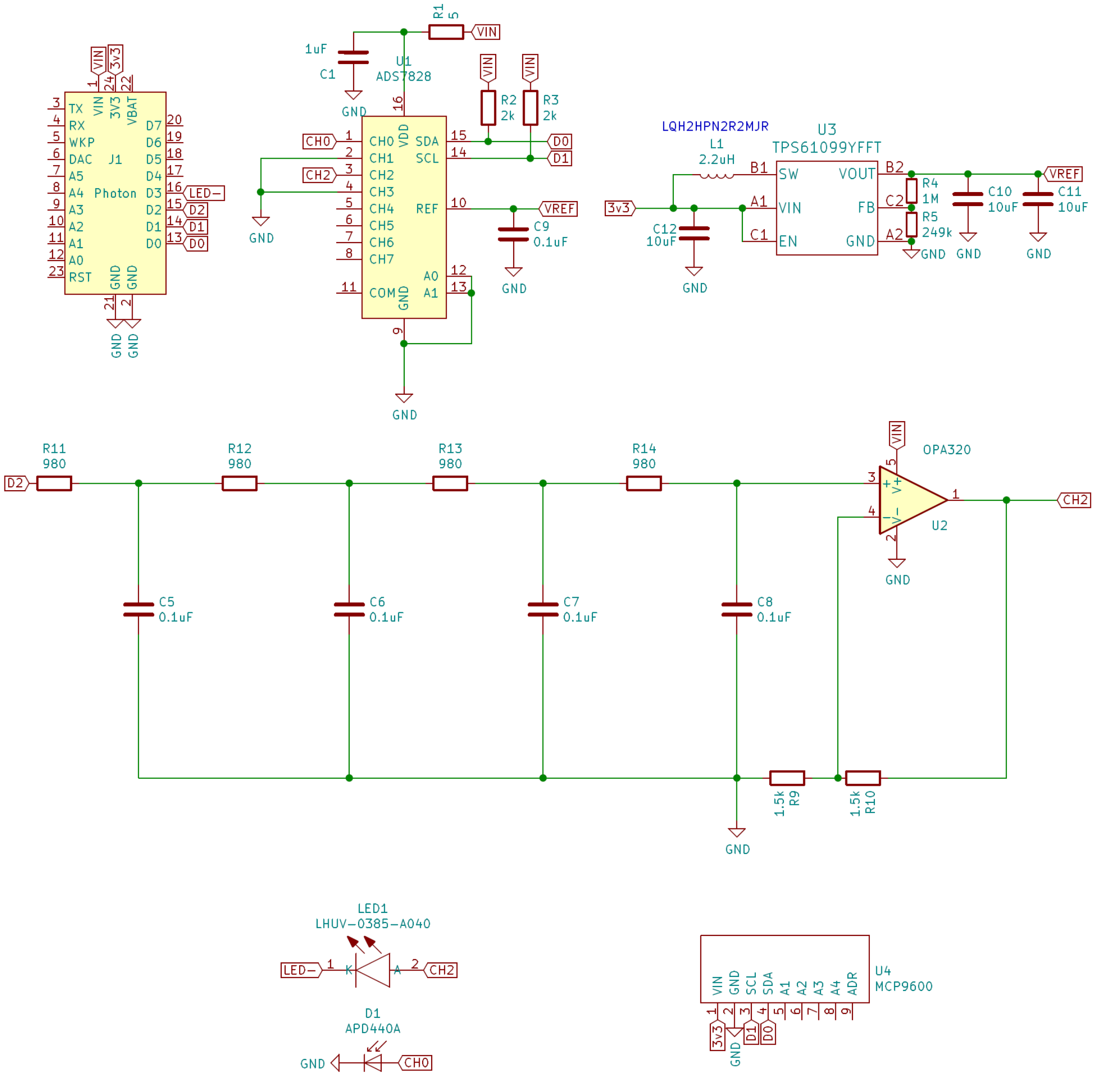

Figure S3: PCB schematics

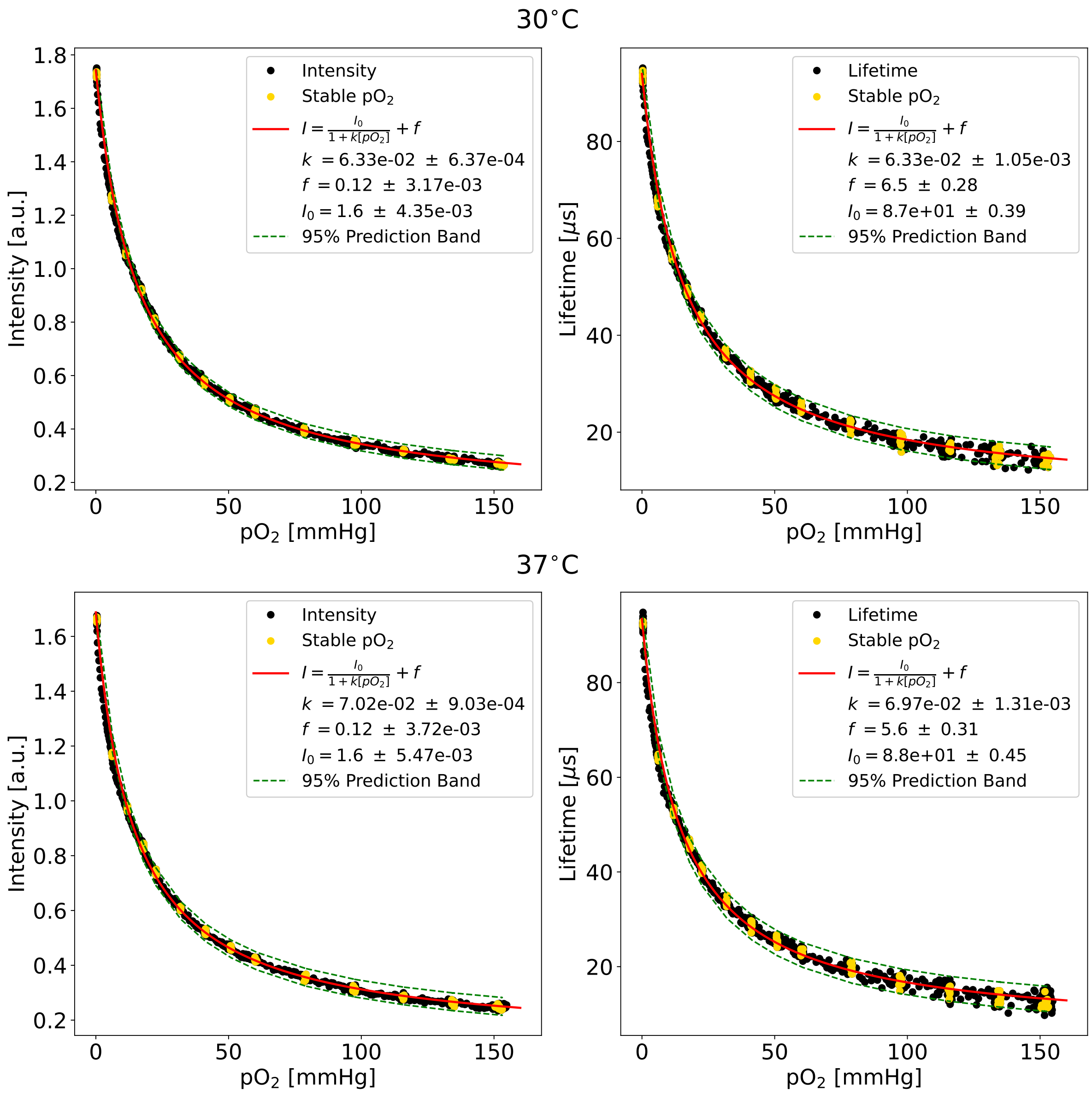

Figure S4: 1D calibration for the sensor from the intensity (left) and the lifetime signal (right) at 30°C and 37°C.

Item	Manufacturer	Manufacturer Part Number	Supplier	Supplier Part Number	Quantity	Price [USD]	Total Price [USD]
1uF capacitance	KEMET	C0402C105K9PACTU	Digi-Key	399-4873-1-ND	1	0.1	0.1
10uF capacitance	Samsung	CL05A106MP5NUNC	Mouser	187-CL05A106MP5NUNC	3	0.18	0.54
0.1uF capacitance	Samsung	CL05F104ZO5NNNC	Digi-Key	1276-1004-1-ND	5	0.1	0.5
Photon microcontroller	Particle	PHOTONH	Digi-Key	1597-1150-ND	1	19	19
2.2uH inductance	Murata	LQH2HPN2R2MJRL	Mouser	81-LQH2HPN2R2MJRL	1	0.4	0.4
LED	Lumileds	LHUV-0385-A040	Mouser	997-LHUV0385A040	1	4.06	4.06
5 Ohm resistor	Vishay Dale	WSC25155R000FEA	Digi-Key	WSCB-5.0CT-ND	1	1.18	1.18
976 Ohm resistor	Yageo	RC0201FR-07976RL	Digi-Key	YAG2853CT-ND	4	0.1	0.4
2 kOhm resistor	Yageo	RC0402FR-072KL	Digi-Key	311-2KLRCT-ND	2	0.1	0.2
1 MOhm resistor	Yageo	RT0603BRD071ML	Mouser	603-RT0603BRD071ML	1	0.31	0.31
249 kOhm resistor	Yageo	RT0603BRD07249KL	Mouser	603-RT0603BRD07249KL	1	0.34	0.34
1.5 kOhm resistor	Panasonic	ERA-2AEB152X	Digi-Key	P1.5KDCCT-ND	2	0.41	0.82
ADC	Texas Instruments	ADS7828EIPWRQ1	Digi-Key	296-24101-1-ND	1	10.14	10.14
Operational amplifier	Texas Instruments	OPA320AQDBVRQ1	Digi-Key	296-45320-1-ND	1	3.03	3.03
Boost converter	Texas Instruments	TPS61099YFFR	Mouser	595-TPS61099YFFR	1	1.12	1.12
Thermocouple amplifier	Adafruit	4101	Digi-Key	1528-4101-ND	1	15.95	15.95
Headers	Amphenol ICC	10129379-909004BLF	Mouser	649-1012937990904BLF	1	0.28	0.28
Photon headers	Sparkfun	PRT-14321	Digi-Key	1568-1650-ND	2	0.81	1.62
BNC adapter	Pomona Electronics	1270	Digi-Key	501-1127-ND	1	20.39	20.39
APD	Thorlabs	APD440A	Thorlabs	APD440A	1	1158.81	1158.81
Multimode fiber coupler	Thorlabs	TH200R5S1B	Thorlabs	TH200R5S1B	1	336.01	336.01
SMA adapter	Thorlabs	SM05SMA	Thorlabs	SM05SMA	1	29.64	29.64
Bulkhead adapter	Thorlabs	HASMA	Thorlabs	HASMA	1	8.36	8.36
Fiber optic mating sleeve	Thorlabs	ADAFCSMA1	Thorlabs	ADAFCSMA1	1	46.59	46.59
Filter	Edmund Optics	39-426	Edmund Optics	39-426	0.01	26	0.26
Kapton tape	3M	5413 Amber 1	Digi-Key	3M541310-ND	0.01	88.62	0.8862
Filter	Edmund Optics	35126	Edmund Optics	35126	0.1	125	12.5
Filter	Edmund Optics	35127	Edmund Optics	35127	0.1	125	12.5
Filter	Edmund Optics	35124	Edmund Optics	35124	0.1	125	12.5
Glass beads	Winsted Precision ball	3200940F62F00A0					0
Thermocouple wire	Omega	EXTT-T20-25	Omega	EXTT-T20-25	0.2	54.53	10.906
Thermocouple socket	Farnell	7086416	Farnell	7086416	1	5.41	5.41

Table S1: Bill of materials.
